# Patterns of Telemedicine Use in Primary Care for People with Dementia in the Post-pandemic Period

**DOI:** 10.1007/s11606-024-08836-1

**Published:** 2024-07-24

**Authors:** Julia Adler-Milstein, Anjali Gopalan, Jie Huang, Christopher Toretsky, Mary Reed

**Affiliations:** 1 Center for Clinical Informatics and Improvement Research, Department of Medicine, University of California, San Francisco, CA USA; 2 Kaiser Permanente Northern California (KPNC) Division of Research, Oakland, CA USA

**Keywords:** telemedicine, dementia care, primary care, utilization, socio-economic status

## Abstract

**Background:**

The pandemic rapidly expanded telemedicine, which has persisted as a widely available primary care modality. The uptake of telemedicine among people with dementia specifically in the primary care setting, who have more complex care needs but also benefit from more accessible primary care, is unknown.

**Objective:**

Among people with dementia, assess uptake of telemedicine-based primary care in the post-pandemic period and determine associations with key socio-demographic characteristics.

**Design:**

Retrospective observational study.

**Subjects:**

People with dementia at UCSF and Kaiser Permanente Northern CA (KPNC) with at least one primary care encounter in pre- (3/1/2019-2/29/2020) or post-COVID (3/1/2021-2/28/2022) periods, post-COVID sample: *N*= 419 individuals (UCSF), *N*=18,037 (KPNC).

**Main Measures:**

Encounter modality: in-person, video telemedicine, or telephone telemedicine. Focal socio-demographic characteristics: age, limited English proficiency, socioeconomic status, driving distance to clinic, and caregiver at encounter.

**Key Results:**

There was a large increase in telemedicine among people with dementia in the post-pandemic period at both sites. At KPNC, those with only in-person primary care visits shrunk from 60.47% (pre) to 26.95% (post). At UCSF, the change was even greater: 98.99% to 35.08%. Across both sites, the only measure significantly associated with use of telemedicine was greater driving distance from home to clinic. At KPNC, those over age 90 were most likely to use telemedicine while patients with limited English proficiency and those with a caregiver at the encounter used telemedicine at lower levels. The relationships were similar at UCSF but not statistically significant.

**Conclusions:**

Telemedicine use is high for people with dementia in the primary care setting in the post-pandemic period. Those with longer drives to clinic and the oldest patients were most likely to use telemedicine, likely due to challenges traveling to appointments. Still, not all people with dementia used telemedicine equally—particularly those with limited English proficiency.

**Supplementary Information:**

The online version contains supplementary material available at 10.1007/s11606-024-08836-1.

## INTRODUCTION

Telemedicine has been widely used in the care of people with dementia for stand-alone or wrap-around interventions (e.g., caregiver support and home monitoring), which are separate from routine primary care.^1–3^ The COVID-19 pandemic caused primary care practices to rapidly expand access to telemedicine visits, and practices have maintained this modality as a care option in the post-pandemic period. However, the large-scale expansion of primary care telemedicine induced by the pandemic was not designed to better enable or support care for this population. Comprehensive primary care for those with dementia is complex and nuanced, with features that may not readily translate to expanded use of telemedicine.^2–4^ Understanding the impact of expanded availability of telemedicine for people with dementia in the primary care setting requires assessing an array of dimensions. However, a foundational question is which people with dementia are using telemedicine as compared to in-person care, as well as the choice of telemedicine modality: phone vs. video visits.

Traditional barriers to telemedicine use—such as older age, limited English proficiency, and lower socio-economic status—likely extend to people with dementia and their caregivers^5–7^ but have not been directly examined.^8–10^ Prior work has examined use of telemedicine during the pandemic and found that preferences, as well as specific barriers (including low technological literacy, lack of technology, inability to follow the email instructions, Internet and bandwidth difficulties, and password difficulties), shaped uptake.^11,12^ In the post-pandemic period in which telemedicine is an option alongside in-person care, it is unknown whether similar factors continue to be relevant.^9,12,13^ Identifying the characteristics associated with use of telemedicine versus in-person care, as well as use of phone vs. video telemedicine, among people with dementia in the primary care setting will provide insight into which factors may be influential. In turn, these insights could signal potential concerns about equitable use as well as suggest how to best target use.

Therefore, in this study, we sought to address these gaps by leveraging data from two large health systems with multiple primary care practices that treat people with dementia—UCSF Health and Kaiser Permanente Northern California (KPNC)—to answer three research questions. For people with dementia in the primary care setting, (1) what modalities of care are utilized and how has this changed pre-to-post pandemic? (2) What socio-demographic characteristics are associated with telemedicine versus in-person modality in the post-pandemic period? (3) Do these vary for phone vs. video? For research questions two and three, we focus on five focal socio-demographic characteristics—age, limited English proficiency, socioeconomic status, driving distance from home to clinic, and presence of a caregiver at the encounter—given the relevance to the dementia population and prior literature suggesting that these factors influence visit modality (Table 1).

**Table 1:** Telemedicine use for People with Dementia: Hypotheses Based on Prior Literature

Demographic characteristic	Hypothesis for telemedicine vs. in person	Hypothesis for phone vs video
Age	**Older people with dementia (PWD)** are **more likely to use telemedicine** visits because it reduces the travel burden (older patients may have greater mobility or mentation challenges that make travel difficult).^8,9,28^	**Older PWD** are **less likely to use video visits** because they have lower digital literacy.^5,29^ **Older PWD** are **more likely to use video visits** if they have a caregiver who can assist them. ^30^
Limited English proficiency	**PWD with limited English proficiency are less likely to use telemedicine visits** due to challenges with translation services. ^5,6,23,28^	**PWD with limited English proficiency** are **less likely to use video visits** because third party interpretation services are more challenging to include in video visits.^24^
Socio-economic status	**PWD with lower SES are less likely to use telemedicine visits** due to lower digital literacy. ^7,28^ Note: among working age, may be the opposite if telemedicine allows them to avoid missing work.^14^	**PWD with lower SES are less likely to use video visits** due to lower digital literacy. ^7,13,29^ **PWD with lower SES** are **less likely to use video visits** due to lack of access to devices with built-in cameras and access to the Internet ^13,28,31,32^
Driving distance from home to clinic	**PWD that must travel farther distances** for an appointment are **more likely to use telemedicine visits** because of the reduced travel burden, in particular disorientation.^8,28^	No difference
Presence of caregiver at encounter	**PWD with a caregiver are more likely to use telemedicine visits** because the caregiver can help with technology requirements.^8,33,34^ **PWD with a caregiver are more likely to use in-person visits** because the caregiver can support travel to the visit.	**PWD with a caregiver are more likely to use video visits** because the caregiver can help with more complex requirements (e.g., computer, smartphone log-in). ^8,33,34^

### Methods

#### Settings

##### USCF Health

The UCSF Division of General Internal Medicine (DGIM) clinics are staffed by 141 primary care providers (including trainees). The primary care population includes ~26,000 patients, predominantly from the immediate seven-county, urban San Francisco Bay Area. Primary care visits are predominantly scheduled over the phone, though self-scheduling via the patient portal was introduced in 2022. In April/May 2020, adults ≥ 65 years old who had upcoming primary care appointments (*n*=1025) were contacted by phone and offered assistance to set up Zoom for video visits. Thirty percent were already connected to the Zoom video platform on their device and were given details about how to use Zoom for their upcoming appointment, 15% were newly connected to the Zoom platform, 33% refused specifically because they preferred a telephone visit or to wait for an in-person visit, 18% had no technology with which to connect to the Zoom platform, and 4% were unable to work the device they did have. Once in-person care was allowed (mid-June 2020), patients could schedule telemedicine or in-person care, based on patient and PCP preference or recommendation by a triage nurse.

*Kaiser Permanente Northern California* is an integrated delivery system with over 1200 primary care providers in 21 medical centers and over 2 million patient-initiated visits per year. KPNC’s service area spans highly urban areas including San Francisco, Oakland, and Sacramento, their surrounding suburbs, as well as rural areas in the California Central Valley. Telephone visits in the ambulatory setting were introduced broadly over a decade ago and video visits were added in 2014.^14^ Patients can access video visits from a computer webcam or through a mobile device (smartphone/tablet) through the KPNC website or mobile Apps (iOS and Android). For the majority of video visits, patients join from a mobile device and speak with their own personal PCP.^14,15^ All ambulatory providers have access to conduct phone visits and to conduct video visits with patients from either a KPNC-enabled computer or mobile device, with telemedicine use integrated directly within the Epic-based electronic health record (EHR). KPNC as a capitated system does not receive different levels of reimbursement by visit modality. Beginning in April 2016, adult patients or their proxies were able to self-schedule a primary care video visit via the patient portal website or mobile app and are asked to choose the type of visit (telephone, video, clinic) with the same set of PCP options and similar schedule availability.

#### Sample

##### Cohort Selection and Study Periods

We created two retrospective cohorts of people with dementia. The pre-COVID period included all individuals with dementia who had a primary care visit between March 1, 2019 through February 29, 2020. The post-COVID cohort included all individuals with dementia with a primary care visit between March 1, 2021 through February 28, 2022. This approach allowed new patients to join the post-COVID cohort—either because they were newly diagnosed with dementia or new to the health system (with dementia). Dementia diagnoses used were defined via ICD-10 codes^16,17^ (Appendix Table 4). People with dementia were defined as those who met one of two criteria: (1) At least one dementia diagnosis code in their problem list or medical history during the period (1/1/2016–2/28/2019 for pre or 1/1/2018–2/28/2021 for post) or (2) at least two dementia diagnosis codes entered at least 4 month apart using the encounter diagnoses, hospital diagnoses, and referrals data sources during the period (1/1/2016–2/28/2019 for pre or 1/1/2018–2/28/2021 for post). See Appendix Figures 3–4 for sample selection details.

##### Encounter Selection 

We extracted all primary care encounters during the study periods that were administered by a physician, nurse practitioner, or physician assistant and had at least one evaluation and management (E&M) visit code (Appendix Table 5). We assigned each visit a modality: in-person, telephone, or video. We used Epic’s visit type name field; any names that contain the word ‘phone’ were considered phone encounters and any names that contain the word ‘video’ were considered video encounters. All remaining encounters were assigned an in-person modality.

#### Measures

##### Socio-demographic characteristics

We defined our five focal characteristics as follows. Age was grouped into categories (<75, 75–79, 80–84, 85–89, and 90+) based on the start of the study period (March 1, 2019, for the pre-COVID cohort and March 1, 2021, for the post-COVID cohort). Limited English proficiency was defined based on the patient’s primary language and was coded as “1” if anything other than English. Socio-economic status was determined from the patient’s zip code and derived using the UCSF Health Atlas.^18^ Driving distance from home to clinic was calculated in miles based on the centroid between the patient’s zip code and the clinic’s zip code. Presence of a caregiver at the encounter was determined from encounter notes. An encounter note that included the term “accompanied by” and one or more of the following terms: caregiver, helper, daughter, son, granddaughter, grandson, daughter-in-law, son-in-law, sister, brother, uncle, aunt, niece, nephew, friend, wife, husband, spouse, and aide was coded as “1.” We captured five additional characteristics to use as controls: sex, race/ethnicity, time since dementia diagnosis, patient portal access, and Charlson Comorbidity Index.

#### Analytic Approach

After producing descriptive statistics to characterize each sample, we aggregated encounter-level data at the individual level within each study period and assigned one of three groupings: (1) all encounters occurred via telemedicine (either phone or video); (2) all encounters occurred in person; or (3) encounters were a mix of telemedicine and in person. We used descriptive statistics to assess changes in modalities pre-to-post pandemic (Research Question 1).

For Research Questions 2 and 3, we used encounter-level logistic regression models to examine associations between our focal characteristics and the use of telemedicine (combining video and phone) versus in-person in the post-pandemic period. Then, among the subset of telemedicine encounters, we used logistic regression to examine associations between our focal characteristics and the use of video versus telephone. All models included the five additional covariates listed above. Socio-economic status and driving distance were both included in the models after confirming they were not correlated (which may not be true in other geographic areas). To visualize levels and differences in composition of modalities within and between the focal characteristics, we then used multinomial logistic regressions to generate the independent associations between visit modality (in-person, video, and telephone) and the focal characteristics. We used a post-estimation command to calculate the adjusted percentages of use of each modality across the focal characteristics via marginal standardization, and present results as bar graphs.^19^

In supplemental analyses using the same modeling approach, we examined additional characteristics that have been shown to be associated with telemedicine use but for which our samples had very little variation due to geographic location. Specifically, we (1) assessed more granular categories of driving distance at KPNC in which the larger sample size supported this breakdown in order to further elucidate the relationship between driving distance and telemedicine use and (2) added two additional covariates—whether the home zip code was in an urban area and a community-level measure of household broadband access using data from the American Community Survey^20^ (encounters where the patient lived in a community with >80% broadband access were coded as “1”).

In all models, standard errors were adjusted for repeat visits by clustering observations by patient with a robust variance estimator. All analysis were completed in parallel at the two sites and approved by each organization’s Institutional Review Board.

## RESULTS

Our sample included 419 (UCSF) and 18,037 (KPNC) people with dementia receiving primary care in the post-COVID period. Sample characteristics differed across the two study sites but stayed largely consistent within site in the pre- vs. post-COVID periods. For example, UCSF had a somewhat younger population and with higher levels of limited English proficiency (one third at UCSF versus ~10% for KPNC). UCSF had just over half of the sample in the highest SES category while this was just under one-quarter in the KPNC sample. Lastly, at the encounter level, about one-quarter of UCSF encounters required individuals to drive more than 5 miles while this was closer to 45% for KPNC encounters (Table 2). Appendix Tables 6–7 report additional patient and encounter summary statistics.

**Table 2:** Sample Demographics and Primary Care Utilization Patterns for UCSF and KPNC Cohorts

	UCSF		KPNC	
	Pre-COVID (3/2019–2/2020)	Post-COVID (3/2021–2/2022)	Pre-COVID (3/2019–2/2020)	Post-COVID (3/2021–2/2022)
Total number of people with dementia	297	419	18339	18037
Patient-level demographics				
Patient age at start of study period.^1^				
<75	76 (25.59%)	107 (25.54%)	3370 (18.38%)	3436 (19.05%)
75–79	52 (17.51%)	70 (16.71%)	2953 (16.1%)	3109 (17.24%)
80–84	62 (20.88%)	87 (20.76%)	4275 (23.31%)	4056 (22.49%)
85–89	67 (22.56%)	89 (21.24%)	4363 (23.79%)	4170 (23.12%)
90+	40 (13.47%)	66 (15.75%)	3378 (18.42%)	3266 (18.11%)
Limited English proficiency				
No	186 (62.63%)	283 (67.54%)	16,459 (89.75%)	15,928 (88.31%)
Yes	111 (37.37%)	136 (32.46%)	1880 (10.25%)	2109 (11.69%)
Neighborhood SES quintile				
1—lowest socio-economic status	1 (0.34%)	1 (0.24%)	746 (4.07%)	747 (4.14%)
2	13 (4.38%)	20 (4.77%)	2592 (14.13%)	2551 (14.14%)
3	49 (16.50%)	56 (13.37%)	4460 (24.32%)	4418 (24.49%)
4	77 (25.93%)	103 (24.58%)	5921 (32.29%)	5917 (32.80%)
5—highest socio-economic status	151 (50.84%)	234 (55.85%)	4300 (23.45%)	4148 (23.00%)
Unknown	6 (2.02%)	5 (1.19%)	320 (1.74%)	256 (1.42%)
Total number of encounters	811	1515	54383	52982
Encounter-level measures				
Distance from home to clinic^2^				
0–<5 miles	627 (77.31%)	1120 (73.93%)	30,497 (56.08%)	29,210 (55.13%)
5+ miles	184 (22.69%)	395 (26.07%)	23,860 (43.87%)	23,739 (44.81%)
Unknown	0 (0.00%)	0 (0.00%)	26 (0.05%)	33 (0.06%)
Presence of caregiver at encounter				
No	34 (4.19%)	105 (6.93%)	16209 (29.81%)	17,196 (32.46%)
Yes	777 (95.81%)	1410 (93.07%)	38174 (70.19%)	35,786 (67.54%)

^1^March 1, 2019, for pre-COVID cohort and March 1, 2021, for post-COVID cohort

^2^We were unable to examine additional levels of this variable to due sample size limitations at UCSF. See Appendix Table 7 for analysis of more granular categories of distance at KPNC

**Figure 1:**
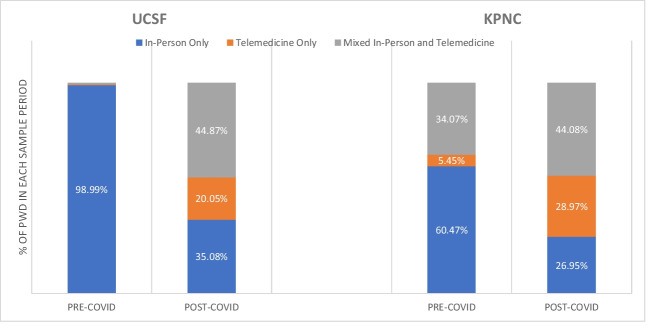
Primary care modality for UCSF and KPNC cohorts of people with dementia. Notes: (1) Pre-COVID (3/2019-2/2020); Post-COVID (3/2021-2/2022), (2) modality group assignment based on type(s) of encounters, (3) telemedicine defined as either telephone visit and/or video visit.

We observed large changes in the pre- vs post-COVID periods in the modality groups (Fig. 1). Overall, KPNC had more telemedicine, particularly phone-based telemedicine (with 22.97% of encounters for people with dementia occurring via phone), in the pre-COVID period while UCSF effectively had no telemedicine in this period (Appendix Table 7). At UCSF, in the pre-COVID period, 98.99% of the sample had only in-person visits and this shrunk to 35.08% in the post-COVID period. The mixed modality group grew from 0.67 to 44.87%, as did the telemedicine only group (from 0.34 to 20.05%). For KPNC in the pre-COVID period, 60.47% of the sample had only in-person visits and this shrunk to 26.95% in the post-COVID period. While the mixed modality group grew from 34.07 to 44.08%, the only telemedicine group markedly expanded from 5.45 to 28.97%.

**Figure 2:**
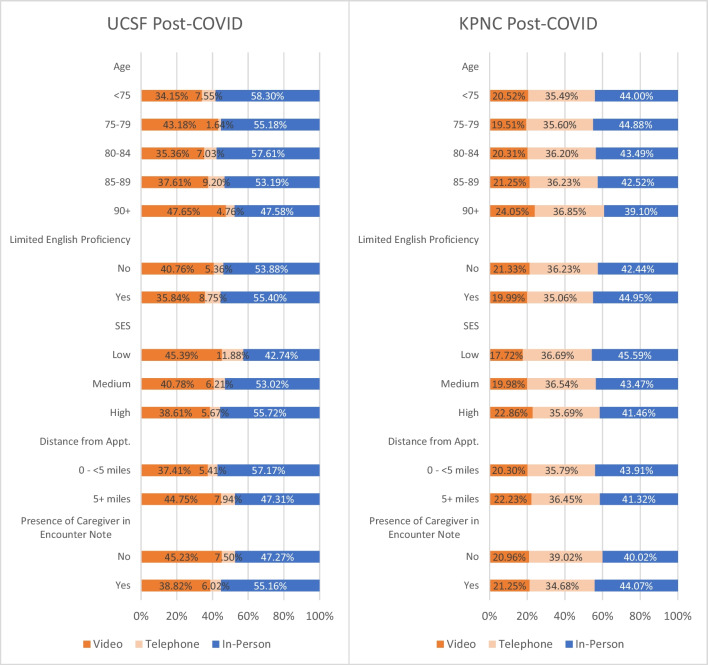
Post-COVID adjusted percentages of each visit modality by focal characteristics.

In post-COVID period logistic regression models, only greater driving distance from home to clinic was significantly associated with odds of selecting telemedicine over in-person encounters at both sites (UCSF 1.52; 95% CI 1.03–2.23; and KPNC 1.11; 95% CI 1.07–1.16) (Table 3; panel 1). When we examined the adjusted percentages of each modality, at UCSF, those who would have needed to drive 5 or more miles to clinic used telemedicine for 52.69% of encounters (44.75% video and 7.94% phone) compared to those with shorter driving distances who used telemedicine for 42.82% of encounters (37.41% video and 5.41% phone). For KPNC, equivalent figures were 58.68% (22.23% video and 36.45% phone) versus 56.10% (20.30% video and 35.79% phone) (Fig. 2). In our sensitivity analysis at KPNC in which we examined more granular categories of driving distance, we found that the odds of telemedicine increased with increased driving distance (compared to <5 miles, 5–<10 miles =1.05 (95% CI 1.00–1.11); 10–<20 miles=1.18 (95% CI 1.10–1.27); 20+ miles=1.30 (95% CI 1.19–1.42) (Appendix Table 10).

**Table 3:** Associations Between Primary Care Modality and Focal Characteristics of People with Dementia: Logistic Regression Results for UCSF and KPNC in Post-COVID Period

		Odds of selecting telemedicine (vs. in person)						Odds of selecting video (vs. telephone) as telemedicine modality					
		UCSF			KPNC			UCSF			KPNC		
		OR	95% CI		OR	95% CI		OR	95% CI		OR	95% CI	
Age group	<75	REF			REF			REF			REF		
	75–79	1.11	0.69	1.80	0.96	0.90	1.04	**4.73**	**1.32**	**16.95**	0.94	0.84	1.04
	80–84	1.05	0.67	1.64	1.02	0.95	1.09	0.99	0.41	2.39	0.96	0.87	1.06
	85–89	1.22	0.75	2.00	1.06	0.99	1.14	0.73	0.24	2.24	1.00	0.90	1.10
	90+	1.11	0.69	1.80	**1.23**	**1.14**	**1.32**	1.72	0.60	4.95	1.11	1.00	1.22
Limited English proficiency	Yes	0.88	0.54	1.42	**0.90**	**0.84**	**0.97**	0.49	0.20	1.23	0.97	0.87	1.08
Neighborhood SES	½ (low)	REF			REF			REF			REF		
	3 (med)	0.67	0.29	1.53	**1.09**	**1.02**	**1.16**	1.52	0.37	6.22	**1.14**	**1.03**	**1.25**
	4/5 (high)	0.59	0.27	1.29	**1.18**	**1.11**	**1.26**	1.93	0.58	6.43	**1.34**	**1.23**	**1.46**
Distance from home to clinic	5+ miles	**1.52**	**1.03**	**2.23**	**1.11**	**1.07**	**1.16**	0.71	0.32	1.58	**1.07**	**1.00**	**1.13**
Presence of caregiver at encounter	Yes	0.71	0.47	1.05	**0.84**	**0.81**	**0.88**	1.27	0.57	2.84	**1.14**	**1.08**	**1.21**

1. Results reflect post-COVID time period only

2. Standard errors adjusted for clustering of patients

3. Bolded results represent significant odds ratios at *p*<0.05

4. Models include the following covariates: sex, race/ethnicity, time since dementia diagnosis, patient portal access, and Charlson Comorbidity Index. Full logistic regression results can be found in Appendix Tables 5–6

5. See Appendix Table 7 for logistic regression results using more granular driving distance categories at KPNC

6. See Appendix Tables 8–9 for logistic regression results including additional measures of Urban Home Zip Code and Percent of Community with Broadband Access in Home Zip Code

While only statistically significant at KPNC, encounters for those aged 90+ versus those under 75 were more likely to be telemedicine versus in-person encounters (UCSF 1.11; 95% CI 0.69–1.80 and KPNC 1.23; 95% CI 1.14–1.32) (Table 3; panel 1). When we examined the adjusted percentages of use of each modality, at UCSF, the adjusted percent of telemedicine encounters for those 90+ was 52.41% (vs. 41.70% for those under age 75) and 60.90% (vs. 56.01%) at KPNC (Fig. 2).

At KPNC, those with higher SES were more likely to select telemedicine encounters (OR 1.18; 95% CI 1.11–1.25, Table 3; panel 1). Those with higher SES were also more likely to select video over phone as the telemedicine modality (OR for moderate SES 1.14; 95% CI 1.03–1.25; OR for high SES 1.34; 95% CI 1.23–1.46, Table 3; panel 2). UCSF had similar results but the odds ratios were not statistically significant. Interestingly, differences in adjusted percentages at KPNC across modalities were relatively small (e.g., 5.14% higher use of video visits in patients living in higher SES neighborhoods than lower SES neighborhoods), and in the opposite direction from those at UCSF (6.78% higher use of video visits in patients living in lower SES neighborhoods than higher SES neighborhoods; Fig. 2).

While only statistically significant at KPNC, encounters for those with limited English proficiency were less likely to be telemedicine versus in-person encounters (UCSF 0.88; 95% CI 0.54–1.42; KPNC 0.90; 95% CI 0.84–0.97, Table 3; panel 1). However, the differences in adjusted percents were modest; for example at UCSF, 44.59% of LEP encounters were telemedicine compared to 46.12% among non-LEP; at KPNC, 55.05% of LEP encounters were telemedicine compared to 57.56% among non-LEP (Fig. 2). Neither site had statistically significant differences between video and phone for LEP encounters.

Lastly, while only statistically significant at KPNC, encounters with a caregiver present were less likely to be telemedicine vs in-person encounters (UCSF 0.71; 95% CI 0.47–1.05; KPNC 0.84; 95% CI 0.81–0.88). At UCSF, the adjusted percent of telemedicine encounters for those with a caregiver present was 44.84% (vs. 52.73%) and 55.93% (vs. 59.98%) at KPNC. While again only statistically significant at KPNC, encounters with a caregiver present were more likely to be video vs telephone encounters (UCSF 1.27; 95% CI 0.57–2.84; KPNC 1.14; 95% CI 1.08–1.21). Full model results are reported in Appendix Tables 8–9.

In supplemental analyses that included urban zip code and community level of broadband access, the directionality and magnitude of the associations between the focal variables and telemedicine vs in-person encounters as well as video vs telephone encounters remained unchanged (Appendix Tables 11–12).

## Discussion

We undertook a large-scale study across two diverse health systems to understand care patterns for people with dementia in the post-COVID period. With telemedicine—both phone and video—now widely available and integrated as care modality options, it is critical to understand how they are being used to care for people with dementia in the primary care setting. Perhaps not surprisingly, we observed a large expansion of telemedicine in the post pandemic period, with more than half of both samples using either all or some telemedicine. When we examined five focal characteristics that address dimensions of equitable access, a mixed picture emerged. Telemedicine is being used more heavily to avoid long drives to in-person encounters. This convenience factor is an obvious and well-known primary benefit of telemedicine but likely disproportionately benefits people with dementia because of the need for caregiver support to travel to appointments as well as confusion or distress when receiving care in an unfamiliar environment. The former is further supported by our finding that presence of a caregiver was associated with greater likelihood of in-person care. Perhaps our most novel finding is that the oldest patients with dementia, those over age 90, were most likely to use telemedicine compared to those under age 75. Results related to SES and limited English proficiency are potential cause for concern and suggest the need to strengthen technology and language support for telemedicine encounters.

When we compared use of the two telemedicine modalities, we were surprised that KPNC had greater use of phone while UCSF had greater use of video and in-person encounters. We suspect that this is due to each organization’s telemedicine investment before the pandemic, with KPNC having heavily invested in phone-based telemedicine and UCSF effectively having no telemedicine. With the severely limited options for in-person care during the COVID-19 pandemic, UCSF prioritized video as the modality to offer as the best alternative, using phone as an option if video was not feasible. Given the persistence of these differences in the post-pandemic period, there is an important opportunity to understand the broad implications for quality. Prior work has called out to the need to further study quality differences—pointing to arguments that support advantages of each modality.^5,21^ Ultimately, the best choice of modality likely depends on the issue(s) being addressed in the encounter. Prior work in geriatrics care suggests that phone encounters appropriately support “history taking, discussion of mood, chronic condition management, medication discussions, dementia management, caregiver support conversations, and discussion of goals of care. Video visits were necessary for dermatologic issues, wound care, leg edema, and other conditions where visual examination adds crucial information.”^9^

As additional evidence emerges on the best approach to match modality to clinical need, it will be even more important to ensure equitable access to telemedicine for people with dementia. Our findings suggest some need for addressing language barriers and a useful first step would be to understand the extent to which these barriers exist on the patient side (e.g., those with LEP do not feel comfortable selecting telemedicine encounters due to concerns about language barriers) and on the health system side (e.g., limited translation services available). Further, in the dementia population in which caregivers are typically involved, it may be that language barriers are less of an issue, which would explain the small magnitude differences. Nonetheless, it is likely worth pursuing efforts to close the observed gaps and prior literature suggests several solutions. These include creating simple instructions in multiple languages for how to use telemedicine platforms and offering language and culturally concordant telemedicine training.^5^ KPNC’s use of medical assistant (MA) rooming prior to video reduced disparities in video visit connection rates by patient language proficiency.^22^ More resource-intensive approaches include formalized outreach to contact patients in their native language, seamless end-to-end (from check-in to visit follow-up) integration of translation services into telemedicine visit technology, and translation of all visit telemedicine setup instructions into all necessary languages.^23,24^

A similar approach may be needed to address barriers to video encounter access (versus phone) for those with lower SES.^25^ One model could be UCSF’s Video Visits for Elders Project (VVEP), which sought to ensure equitable access to video encounters. One to 2 weeks before their scheduled appointment, VVEP team members contacted patients in their preferred language, as documented in the EHR. The team then (1) communicated UCSF’s recent transition to video visits, (2) assessed the patient’s access to an electronic device, (3) offered a tutorial on downloading Zoom and initiating a connection, and (4) provided any additional information (ex. Meeting ID). Among patients that accepted VVEP assistance, 77% successfully downloaded Zoom.^26^ There is potential for other organizations to develop and implement similar models; for example, CMS’s newly released Guiding an Improved Dementia Experience (GUIDE) Model could be tailored to telemedicine. Key elements of the GUIDE model that could integrate telemedicine in ways that promote equitable uptake include defining standardized approaches to dementia caregiving, supporting further innovation in care delivery, screening for health-related social needs, and providing education and support to caregivers.^27^

Our study has important limitations to consider. First, UCSF had a small sample of people with dementia, which likely contributed to underpowered results. The very large sample at KPNC allows us to assess where relationships (such as between LEP and use of telemedicine) are consistent with those in the smaller UCSF sample in terms of directionality and magnitude. Further, given that dementia is commonly underdiagnosed in primary care settings, some patients with dementia were likely missed at both sites; we tried to minimize this by using a long look-back period to capture dementia diagnoses. In addition, given the multiple primary care practices included in each study site, we were not able to account for differences in scheduling practices, PCP availability, or other clinic- and site-level factors driving selection of modality. Where relevant, we described known factors, such as UCSF’s outreach program to ensure video access. Similarly, we had no way to capture when patient preference versus clinician preference versus availability of different modality options drove modality selection. We also lacked systematic data on reason for visit and whether the reason directly related to dementia, as well as information on a patient’s functional status and level of education. Such information would have been valuable to better understand the use of telemedicine in this population and represents an important priority for future work. Lastly, there is potential that our method to capture caregiver presence at encounters was biased. Specifically, notes from in-person encounters may be more likely to document caregiver presence, making it important to refine this measure in future work in order to understand whether caregivers better facilitate one visit modality over another. Our work therefore offers an important starting point for understanding the associations between characteristics and encounter modality, and future work on the mechanisms will create a full understanding.

## Conclusion

Using multi-site data, we conducted a novel assessment of use of telemedicine in the primary care setting for people with dementia. Overall, we found a large expansion of telemedicine in the post-pandemic period, suggesting sustained use and pointing to the need to understand the implications for access and quality. Interestingly, use of video varied substantially between sites, suggesting that choice of specific modality is still variable and in need of better evidence to guide optimal selection. We also found that a key benefit of telemedicine—reduced need for driving long distances to the clinic—is being realized. With respect to equitable access, our results reveal that the oldest patients were most likely to use telemedicine. However, there are some concerns related to equitable access for those with limited English proficiency and lower SES. Interventions to address these disparities will help ensure that all people with dementia can take advantage of the benefits of telemedicine.

## Supplementary Information

Below is the link to the electronic supplementary material.

Supplementary file1 (DOCX 94.3 KB)
