# Supplementary material for: Patterns of Telemedicine Use in Primary Care for People with Dementia in the Post-pandemic Period
Source: J Gen Intern Med. 2024 Jul 24;39(15):2895–903. doi: 10.1007/s11606-024-08836-1 (PMC11576693; doi:10.1007/s11606-024-08836-1)
Supplement: Supplementary file 1 — Supplementary file1 (DOCX 94.3 KB) [file 11606_2024_8836_MOESM1_ESM.docx]

**APPENDIX**

**Table 1. ICD-10 Codes Used to Define Persons with Dementia**

| **ICD-10** | **ICD-10 Description** |
| --- | --- |
| F01.5 | Vascular dementia* |
| F01.50 | Vascular dementia without behavioral disturbance |
| F01.51 | Vascular dementia with behavioral disturbance |
| G30 | Alzheimer's disease* |
| G30.0 | Alzheimer's disease with early onset |
| G30.1 | Alzheimer's disease with late onset |
| G30.8 | Other Alzheimer's disease |
| G30.9 | Alzheimer's disease, unspecified |
| G31.0 | Frontotemporal dementia* |
| G31.01 | Pick's disease |
| G31.09 | Other frontotemporal dementia |
| G31.83 | Dementia with Lewy bodies |
| F02.80 | Dementia in other diseases classified elsewhere without behavioral disturbance |
| F02.81 | Dementia in other diseases classified elsewhere with behavioral disturbance |
| F03.90 | Unspecified dementia without behavioral disturbance |
| F03.91 | Unspecified dementia with behavioral disturbance |

* F01.5, G30, and G31.0 were added for the current study based on expert input while the other codes were derived from prior work.

**Table 2. CPT Codes Used for Primary Care Encounters**

| **CPT Code** | **Description** |  |  |  |  |  |  |
| --- | --- | --- | --- | --- | --- | --- | --- |
| 99201 | Office Outpt New 10 Min | |  |  |  |  |  |
| 99202 | Office Outpt New 20 Min | |  |  |  |  |  |
| 99203 | Office Outpt New 30 Min | |  |  |  |  |  |
| 99204 | Office Outpt New 45 Min | |  |  |  |  |  |
| 99205 | Office Outpt New 60 Min | |  |  |  |  |  |
| 99211 | Office O/P Est 5 Min | |  |  |  |  |  |
| 99212 | Office Outpt Est 10 Min | |  |  |  |  |  |
| 99213 | Office Outpt Est15 Min | |  |  |  |  |  |
| 99214 | Office Outpt Est 25 Min | |  |  |  |  |  |
| 99215 | Office Outpt Est 40 Min | |  |  |  |  |  |
| 99241 | Office Consltj 15 Min | |  |  |  |  |  |
| 99242 | Office Consltj 30 Min | |  |  |  |  |  |
| 99243 | Office Consltj 40 Min | |  |  |  |  |  |
| 99244 | Office Consltj 60 Min | |  |  |  |  |  |
| 99245 | Office Consltj 80 Min | |  |  |  |  |  |
| 99441 | Physician Telephone Evaluation 5-10 Min | | |  |  |  |  |
| 99442 | Physician Telephone Evaluation 11-20 Min | | |  |  |  |  |
| 99443 | Physician Telephone Evaluation 21-30 Min | | |  |  |  |  |
| 99385 | 1St Preventive Medicine New Patient Age 18-39Yrs | | | |  |  |  |
| 99386 | 1St Preventive Medicine New Patient Age 40-64Yrs | | | |  |  |  |
| 99387 | 1St Preventive Medicine New Patient Age 65Yrs&> | | | |  |  |  |
| 99395 | Periodic Preventive Med Est Patient Age 18-39Yrs | | |  |  |  |  |
| 99396 | Periodic Preventive Med Est Patient Age 40-64Yrs | | |  |  |  |  |
| 99397 | Periodic Preventive Med Est Patient Age 65Yrs&> | | |  |  |  |  |
| G0402 | Initial Preventive Physical Examination; Face-To-Face Visit, Services Limited To  New Beneficiary During The First 12 Months Of Medicare Enrollment | | | | | | |
| G0438 | Annual Wellness Visit, Includes A Personalized Prevention Plan Of Service First Visit | | | | | |  |
| G0439 | Annual Wellness Visit, Includes A Personalized Prevention Plan Of Service Subsequent Visit | | | | | |  |

**Figure 1. KPNC Sample Selection Diagram**

**Figure 2. UCSF Sample Selection Diagram**

**Table 3. Additional Sample Demographics for UCSF and KPNC Cohorts**

|  | UCSF | | KPNC | |
| --- | --- | --- | --- | --- |
|  | Pre-COVID  (3/2019 – 2/2020) | Post-COVID (3/2021 – 2/2022) | Pre-COVID  (3/2019 – 2/2020) | Post-COVID (3/2021 – 2/2022) |
| **Total number of people with dementia** | 297 | 419 | 18339 | 18037 |
| **PATIENT-LEVEL DEMOGRAPHICS** |  |  |  |  |
| **Sex** |  |  |  |  |
| Female | 197 (66.33%) | 271 (64.68%) | 11865 (64.70%) | 11710 (64.92%) |
| Male | 100 (33.67%) | 148 (35.32%) | 6474 (35.30%) | 6327 (35.08%) |
| **Race/Ethnicity** |  |  |  |  |
| Asian | 122 (41.08%) | 167 (39.86%) | 2538 (13.84%) | 2667 (14.79%) |
| Black or African American | 35 (11.78%) | 36 (8.59%) | 1629 (8.88%) | 1611 (8.93%) |
| Latinx | 23 (7.74%) | 38 (9.07%) | 2695 (14.70%) | 2766 (15.34%) |
| White | 102 (34.34%) | 154 (36.75%) | 10441 (56.93%) | 10031 (55.61%) |
| Other/Multi-Race | 14 (4.71%) | 24 (5.73%) | 1035 (5.64%) | 961 (5.33%) |
| Unknown/Declined | 1 (0.34%) | 0 (0.00%) | 1 (0.01%) | 1 (0.01%) |
| **Total number of encounters** | 811 | 1515 | 54383 | 52982 |
| **ENCOUNTER-LEVEL MEASURES** |  |  |  |  |
| **Time Since Diagnosis** |  |  |  |  |
| 0 to <1 years | 71 (8.75%) | 170 (11.22%) | 7533 (13.85%) | 6898 (13.02%) |
| 1 to <2 years | 184 (22.69%) | 339 (22.38%) | 13311 (24.48%) | 12505 (23.60%) |
| 2 to <3 years | 231 (28.48%) | 339 (22.38%) | 13299 (24.45%) | 12450 (23.50%) |
| 3+ years | 325 (40.07%) | 667 (44.03%) | 20240 (37.22%) | 21129 (39.88%) |
| **Patient Portal Access** |  |  |  |  |
| No | 175 (21.58%) | 89 (5.87%) | 11237 (20.66%) | 947 (1.79%) |
| Yes | 636 (78.42%) | 1426 (94.13%) | 43146 (79.34%) | 52035 (98.21%) |
| **Charlson Comorbidity Index** |  |  |  |  |
| 0 | 31 (3.82%) | 38 (2.51%) | 592 (1.09%) | 2944 (5.56%) |
| 1 | 180 (22.19%) | 354 (23.37%) | 5008 (9.21%) | 5460 (10.31%) |
| 2 | 176 (21.70%) | 281 (18.55%) | 9262 (17.03%) | 8412 (15.88%) |
| 3 | 124 (15.29%) | 243 (16.04%) | 8689 (15.98%) | 7897 (14.91%) |
| 4+ | 300 (36.99%) | 599 (39.54%) | 30832 (56.69%) | 28269 (53.36%) |
| **Urban Home Zip Code** |  |  |  |  |
| No | 8 (0.99%) | 13 (0.86%) | 2100 (3.86%) | 2014 (3.80%) |
| Yes | 803 (99.01%) | 1502 (99.14%) | 52260 (96.10%) | 50936 (96.14%) |
| Unknown | 0 (0.00%) | 0 (0.00%) | 23 (0.04%) | 32 (0.06%) |
| **Percent of Community with Broadband Access in Home Zip Code** |  |  |  |  |
| Mean (SD) | 90.35 (5.15) | 91.69 (4.22) | 92.19 (4.14) | 92.17 (4.16) |
| **Distance from Home to Clinic (Detail)** |  |  |  |  |
| 0 - <5 miles | 627 (77.31%) | 1120 (73.93%) | 30497 (56.08%) | 29210 (55.13%) |
| 5 - <10 miles | 44 (5.43%) | 101 (6.67%) | 14857 (27.32%) | 14690 (27.73%) |
| 10 - <20 miles | 86 (10.60%) | 174 (11.49%) | 5858 (10.77%) | 5870 (11.08%) |
| 20+ miles | 54 (6.66%) | 120 (7.92%) | 3145 (5.78%) | 3179 (6.00%) |
| Unknown | 0 (0.00%) | 0 (0.00%) | 26 (0.05%) | 33 (0.06%) |

**Table 4. Primary Care Utilization Patterns for UCSF and KPNC Cohorts**

|  | UCSF | | KPNC | |
| --- | --- | --- | --- | --- |
|  | Pre-COVID  (3/2019 – 2/2020) | Post-COVID (3/2021 – 2/2022) | Pre-COVID  (3/2019 – 2/2020) | Post-COVID (3/2021 – 2/2022) |
| **Total number of people with dementia** | 297 | 419 | 18339 | 18037 |
| **UTILIZATION** |  |  |  |  |
| **Number of Primary Care Visits^1^** |  |  |  |  |
| 1 | 103 (34.68%) | 95 (22.67%) | 5586 (30.46%) | 5690 (31.55%) |
| 2 | 60 (20.20%) | 86 (20.53%) | 4354 (23.74%) | 4345 (24.09%) |
| 3 | 54 (18.18%) | 58 (13.84%) | 3004 (16.38%) | 2854 (15.82%) |
| 4 | 34 (11.45%) | 61 (14.56%) | 1974 (10.76%) | 1857 (10.30%) |
| 5+ | 46 (15.51%) | 119 (28.38%) | 3421 (18.65%) | 3291 (18.25%) |
|  | UCSF |  | KPNC |  |
|  | Pre-COVID (3/2019 – 2/2020) | Post-COVID (3/2021 – 2/2022) | Pre-COVID (3/2019 – 2/2020) | Post-COVID (3/2021 – 2/2022) |
| **Total number of encounters** | 811 | 1515 | 54383 | 52982 |
| **Visit Modality** |  |  |  |  |
| In-Person | 805 (99.26%) | 827 (54.59%) | 41664 (76.61%) | 22645 (42.74%) |
| Telephone | 0 (0.00%) | 92 (6.07%) | 12491 (22.97%) | 19125 (36.10%) |
| Video | 6 (0.74%) | 596 (39.34%) | 228 (0.42%) | 11212 (21.16%) |

Notes

1. Primary care visits were visits administered by a physician, nurse practitioner, or physician assistant in primary care departments and which were associated with an E&M visit code.

**Table 5. Full Logistic Regression Results of Telemedicine (Telephone or Video) vs. In-person Encounters in Post-COVID Period.**

|  |  | **ODDS OF SELECTING TELEMEDICINE (vs. IN PERSON)** | | | | | |
| --- | --- | --- | --- | --- | --- | --- | --- |
|  |  | UCSF | | | KPNC | | |
|  |  | OR | 95% CI | | OR | 95% CI | |
| Age group | <75 | REF | | | REF | | |
|  | 75-79 | 1.11 | 0.69 | 1.80 | 0.96 | 0.90 | 1.04 |
|  | 80-84 | 1.05 | 0.67 | 1.64 | 1.02 | 0.95 | 1.09 |
|  | 85-89 | 1.22 | 0.75 | 2.00 | 1.06 | 0.99 | 1.14 |
|  | 90+ | 1.58 | 0.94 | 2.68 | **1.23** | **1.14** | **1.32** |
| Limited English Proficiency | Yes | 0.88 | 0.54 | 1.42 | **0.90** | **0.84** | **0.97** |
| Neighborhood SES | 1/2 (low) | REF | | | REF | | |
|  | 3 (medium) | 0.67 | 0.29 | 1.53 | **1.09** | **1.02** | **1.16** |
|  | 4/5 (high) | 0.59 | 0.27 | 1.29 | **1.18** | **1.11** | **1.26** |
| Distance from Home to Clinic | 5+ miles | **1.52** | **1.03** | **2.23** | **1.11** | **1.07** | **1.16** |
| Presence of Caregiver at Encounter | Yes | 0.71 | 0.47 | 1.05 | **0.84** | **0.81** | **0.88** |
| Sex | Female | **1.42** | **1.00** | **2.01** | **1.11** | **1.06** | **1.16** |
| Race/Ethnicity | White | REF | | | REF | | |
|  | Asian | 0.73 | 0.43 | 1.24 | **1.12** | **1.04** | **1.20** |
|  | Black or African American | 0.60 | 0.31 | 1.14 | **1.26** | **1.17** | **1.36** |
|  | Latinx | 0.89 | 0.46 | 1.72 | 1.03 | 0.96 | 1.10 |
|  | Other/Multi-Race | 0.66 | 0.30 | 1.45 | 1.04 | 0.95 | 1.15 |
| Time from Dementia Diagnosis (years) | 0 | REF | | | REF | | |
|  | 1 | 1.12 | 0.68 | 1.85 | 0.96 | 0.90 | 1.03 |
|  | 2 | 0.66 | 0.38 | 1.12 | 1.01 | 0.95 | 1.09 |
|  | 3+ | 0.80 | 0.47 | 1.35 | **1.09** | **1.02** | **1.16** |
| History of Portal Use | Yes | 1.49 | 0.78 | 2.84 | **0.55** | **0.46** | **0.66** |
| Charlson Comorbidity Index | 0 or 1 | REF | | | REF | | |
|  | 2 | 1.02 | 0.64 | 1.62 | **0.89** | **0.82** | **0.95** |
|  | 3 | 0.89 | 0.55 | 1.44 | 1.00 | 0.93 | 1.08 |
|  | 4+ | 1.11 | 0.71 | 1.71 | **1.15** | **1.09** | **1.22** |

Notes:

1. Results reflect post-COVID time period only.
2. Standard errors adjusted for clustering of patients.
3. Bolded results represent significant odds ratios at p<0.05.
4. Modality group sample sizes: UCSF (Telemedicine: n=688; DOV: n=827), KPNC (Telemedicine: n=30,337; DOV: n=22,645).

**Table 6. Full Logistic Regression Results of Telephone vs. Video Encounters in Post-COVID Period.**

|  |  | **ODDS OF SELECTING VIDEO (vs. TELEPHONE) AS MODALITY** | | | | | |
| --- | --- | --- | --- | --- | --- | --- | --- |
|  |  | UCSF | | | KPNC | | |
|  |  | OR | 95% CI | | OR | 95% CI | |
| Age group | <75 | REF | | | REF | | |
|  | 75-79 | **4.73** | **1.32** | **16.95** | 0.94 | 0.84 | 1.04 |
|  | 80-84 | 0.99 | 0.41 | 2.39 | 0.96 | 0.87 | 1.06 |
|  | 85-89 | 0.73 | 0.24 | 2.24 | 1.00 | 0.90 | 1.10 |
|  | 90+ | 1.72 | 0.60 | 4.95 | 1.11 | 1.00 | 1.22 |
| Limited English Proficiency | Yes | 0.49 | 0.20 | 1.23 | 0.97 | 0.87 | 1.08 |
| Neighborhood SES | 1/2 (low) | REF | | | REF | | |
|  | 3 (medium) | 1.52 | 0.37 | 6.22 | **1.14** | **1.03** | **1.25** |
|  | 4/5 (high) | 1.93 | 0.58 | 6.43 | **1.34** | **1.23** | **1.46** |
| Distance from Home to Clinic | 5+ miles | 0.71 | 0.32 | 1.58 | **1.07** | **1.00** | **1.13** |
| Presence of Caregiver at Encounter | Yes | 1.27 | 0.57 | 2.84 | **1.14** | **1.08** | **1.21** |
| Sex | Female | 0.92 | 0.41 | 2.07 | **1.10** | **1.03** | **1.17** |
| Race/Ethnicity | White | REF | | | REF | | |
|  | Asian | **3.66** | **1.40** | **9.54** | **1.36** | **1.23** | **1.50** |
|  | Black or African American | 0.86 | 0.28 | 2.68 | **0.78** | **0.70** | **0.88** |
|  | Latinx | 2.28 | 0.57 | 9.08 | 0.99 | 0.89 | 1.09 |
|  | Other/Multi-Race | 0.61 | 0.13 | 2.92 | 0.92 | 0.80 | 1.06 |
| Time from Dementia Diagnosis (years) | 0 | REF | | | REF | | |
|  | 1 | 2.32 | 0.96 | 5.59 | 0.96 | 0.86 | 1.06 |
|  | 2 | 1.53 | 0.56 | 4.18 | 1.10 | 0.99 | 1.22 |
|  | 3+ | 1.17 | 0.47 | 2.91 | 1.02 | 0.93 | 1.13 |
| History of Portal Use | Yes | **15.18** | **3.55** | **64.87** | **1.31** | **1.08** | **1.59** |
| Charlson Comorbidity Index | 0 or 1 | REF | | | REF | | |
|  | 2 | 1.61 | 0.43 | 6.10 | **0.63** | **0.56** | **0.70** |
|  | 3 | 1.38 | 0.44 | 4.31 | **0.65** | **0.58** | **0.72** |
|  | 4+ | 0.82 | 0.33 | 2.04 | **0.58** | **0.53** | **0.63** |

Notes:

1. Results reflect post-COVID time period only.
2. Standard errors adjusted for clustering of patients.
3. Bolded results represent significant odds ratios at p<0.05.

**Table 7. Full Logistic Regression Results of Telemedicine (Telephone or Video) vs. In-person Encounters in Post-COVID Period: More Granular Driving Distance Categories at KPNC.**

|  |  | **ODDS OF SELECTING TELEMEDICINE (vs. IN PERSON)** | | |
| --- | --- | --- | --- | --- |
|  |  | KPNC | | |
|  |  | OR | 95% CI | |
| Age group | <75 | REF | | |
|  | 75-79 | 0.97 | 0.90 | 1.04 |
|  | 80-84 | 1.02 | 0.96 | 1.09 |
|  | 85-89 | 1.06 | 0.99 | 1.13 |
|  | 90+ | **1.22** | **1.14** | **1.31** |
| Limited English Proficiency | Yes | **0.90** | **0.84** | **0.97** |
| Neighborhood SES | 1/2 (low) | REF | | |
|  | 3 (medium) | **1.09** | **1.02** | **1.16** |
|  | 4/5 (high) | **1.19** | **1.12** | **1.27** |
| Distance from Home to Clinic | 5+ miles | REF | | |
|  | 5-<10 miles | **1.05** | **1.00** | **1.11** |
|  | 10-<20 miles | **1.18** | **1.10** | **1.27** |
|  | 20+ miles | **1.30** | **1.19** | **1.42** |
| Presence of Caregiver at Encounter | Yes | **0.84** | **0.81** | **0.88** |
| Sex | Female | **1.11** | **1.06** | **1.16** |
| Race/Ethnicity | White | REF | | |
|  | Asian | **1.12** | **1.04** | **1.20** |
|  | Black or African American | **1.27** | **1.17** | **1.36** |
|  | Latinx | 1.03 | 0.97 | 1.11 |
|  | Other/Multi-Race | 1.05 | 0.95 | 1.15 |
| Time from Dementia Diagnosis (years) | 0 | REF | | |
|  | 1 | 0.96 | 0.90 | 1.03 |
|  | 2 | 1.01 | 0.94 | 1.09 |
|  | 3+ | **1.08** | **1.01** | **1.16** |
| History of Portal Use | Yes | **0.56** | **0.47** | **0.67** |
| Charlson Comorbidity Index | 0 or 1 | REF | | |
|  | 2 | **0.89** | **0.82** | **0.95** |
|  | 3 | 1.00 | 0.93 | 1.08 |
|  | 4+ | **1.15** | **1.09** | **1.22** |

Notes:

1. Results reflect post-COVID time period only.
2. Standard errors adjusted for clustering of patients.
3. Bolded results represent significant odds ratios at p<0.05.
4. Modality group sample sizes: KPNC (Telemedicine: n=30,337; DOV: n=22,645).

**Table 8. Full Logistic Regression Results of Telemedicine (Telephone or Video) vs. In-person Encounters in Post-COVID Period: Including Measures of Urban Home Zip Code and Level of Community Broadband.**

|  |  | **ODDS OF SELECTING TELEMEDICINE (vs. IN PERSON)** | | | | | |
| --- | --- | --- | --- | --- | --- | --- | --- |
|  |  | UCSF | | | KPNC | | |
|  |  | OR | 95% CI | | OR | 95% CI | |
| Age group | <75 | REF | | | REF | | |
|  | 75-79 | 1.11 | 0.69 | 1.80 | 0.97 | 0.90 | 1.04 |
|  | 80-84 | 1.07 | 0.68 | 1.68 | 1.02 | 0.95 | 1.09 |
|  | 85-89 | 1.24 | 0.76 | 2.02 | 1.06 | 1.00 | 1.14 |
|  | 90+ | 1.56 | 0.92 | 2.65 | **1.23** | **1.14** | **1.32** |
| Limited English Proficiency | Yes | 0.90 | 0.56 | 1.46 | **0.90** | **0.84** | **0.98** |
| Neighborhood SES | 1/2 (low) | REF | | | REF | | |
|  | 3 (medium) | 0.72 | 0.31 | 1.69 | **1.08** | **1.01** | **1.15** |
|  | 4/5 (high) | 0.60 | 0.27 | 1.31 | **1.16** | **1.09** | **1.23** |
| Distance from Home to Clinic | 5+ miles | **1.55** | **1.04** | **2.30** | **1.13** | **1.08** | **1.18** |
| Presence of Caregiver at Encounter | Yes | 0.70 | 0.47 | 1.05 | **0.85** | **0.81** | **0.88** |
| Sex | Female | 1.39 | 0.98 | 1.98 | **1.11** | **1.07** | **1.16** |
| Race/Ethnicity | White | REF | | | REF | | |
|  | Asian | 0.72 | 0.42 | 1.24 | **1.11** | **1.04** | **1.19** |
|  | Black or African American | 0.58 | 0.31 | 1.11 | **1.25** | **1.16** | **1.35** |
|  | Latinx | 0.86 | 0.44 | 1.69 | 1.03 | 0.96 | 1.10 |
|  | Other/Multi-Race | 0.64 | 0.29 | 1.43 | 1.04 | 0.94 | 1.14 |
| Time from Dementia Diagnosis (years) | 0 | REF | | | REF | | |
|  | 1 | 1.10 | 0.66 | 1.81 | 0.96 | 0.90 | 1.03 |
|  | 2 | 0.65 | 0.38 | 1.12 | 1.02 | 0.95 | 1.09 |
|  | 3+ | 0.80 | 0.47 | 1.35 | **1.09** | **1.02** | **1.17** |
| History of Portal Use | Yes | 1.38 | 0.72 | 2.63 | **0.55** | **0.46** | **0.65** |
| Charlson Comorbidity Index | 0 or 1 | REF | | | REF | | |
|  | 2 | 1.03 | 0.65 | 1.64 | **0.89** | **0.82** | **0.95** |
|  | 3 | 0.92 | 0.57 | 1.48 | 1.00 | 0.93 | 1.08 |
|  | 4+ | 1.13 | 0.73 | 1.75 | **1.15** | **1.09** | **1.22** |
| Urban Home Zip Code | Yes | 2.39 | 0.95 | 6.04 | **1.14** | **1.01** | **1.29** |
| Percent of Community with Broadband Access in Home Zip Code | >80% | 1.45 | 0.65 | 3.26 | **1.29** | **1.12** | **1.50** |

Notes:

1. Results reflect post-COVID time period only.
2. Standard errors adjusted for clustering of patients.
3. Bolded results represent significant odds ratios at p<0.05.
4. Modality group sample sizes: UCSF (Telemedicine: n=688; DOV: n=827), KPNC (Telemedicine: n=30,337; DOV: n=22,645).

**Table 9. Full Logistic Regression Results of Telephone vs. Video Encounters in Post-COVID Period at KPNC: Including Measures of Urban Home Zip Code and Level of Community Broadband.**

|  |  | **ODDS OF SELECTING VIDEO (vs. TELEPHONE) AS MODALITY** | | |
| --- | --- | --- | --- | --- |
|  |  | KPNC | | |
|  |  | OR | 95% CI | |
| Age group | <75 | REF | | |
|  | 75-79 | 0.94 | 0.84 | 1.04 |
|  | 80-84 | 0.96 | 0.87 | 1.06 |
|  | 85-89 | 1.00 | 0.90 | 1.10 |
|  | 90+ | 1.11 | 1.00 | 1.23 |
| Limited English Proficiency | Yes | 0.97 | 0.87 | 1.08 |
| Neighborhood SES | 1/2 (low) | REF | | |
|  | 3 (medium) | **1.14** | **1.04** | **1.26** |
|  | 4/5 (high) | **1.33** | **1.22** | **1.45** |
| Distance from Home to Clinic | 5+ miles | **1.08** | **1.01** | **1.15** |
| Presence of Caregiver at Encounter |  | **1.14** | **1.08** | **1.21** |
| Sex | Female | **1.10** | **1.03** | **1.17** |
| Race/Ethnicity | White | REF | | |
|  | Asian | **1.35** | **1.23** | **1.50** |
|  | Black or African American | **0.78** | **0.70** | **0.87** |
|  | Latinx | 0.98 | 0.89 | 1.08 |
|  | Other/Multi-Race | 0.92 | 0.80 | 1.05 |
| Time from Dementia Diagnosis (years) | 0 | REF | | |
|  | 1 | 0.96 | 0.86 | 1.06 |
|  | 2 | 1.10 | 0.99 | 1.22 |
|  | 3+ | 1.02 | 0.93 | 1.13 |
| History of Portal Use | Yes | **1.31** | **1.08** | **1.59** |
| Charlson Comorbidity Index | 0 or 1 | REF | | |
|  | 2 | **0.63** | **0.56** | **0.70** |
|  | 3 | **0.65** | **0.58** | **0.72** |
|  | 4+ | **0.58** | **0.53** | **0.63** |
| Urban Home Zip Code | Yes | 1.15 | 0.97 | 1.36 |
| Percent of Community with Broadband Access in Home Zip Code | >80% | **1.01** | **0.81** | **1.26** |

Notes:

1. Results reflect post-COVID time period only.
2. Standard errors adjusted for clustering of patients.
3. Bolded results represent significant odds ratios at p<0.05.
